# Toddaculin, Isolated from of *Toddalia asiatica* (L.) Lam., Inhibited Osteoclastogenesis in RAW 264 Cells and Enhanced Osteoblastogenesis in MC3T3-E1 Cells

**DOI:** 10.1371/journal.pone.0127158

**Published:** 2015-05-18

**Authors:** Akio Watanabe, Momochika Kumagai, Takashi Mishima, Junya Ito, Yurika Otoki, Teppei Harada, Tsuyoshi Kato, Mikihiko Yoshida, Misora Suzuki, Izumi Yoshida, Kazuhiro Fujita, Masatoshi Watai, Kiyotaka Nakagawa, Teruo Miyazawa

**Affiliations:** 1 Food Function Research Team, Saito Laboratory, Japan Food Research Laboratories, Ibaraki, Osaka, Japan; 2 Food and Biodynamic Chemistry Laboratory, Graduate School of Agricultural Science, Tohoku University, Sendai, Miyagi, Japan; 3 Section of Applied Testing, Tama Laboratory, Japan Food Research Laboratories, Tama, Tokyo, Japan; 4 Section of Nutraceutical Analysis, Saito Laboratory, Japan Food Research Laboratories, Ibaraki, Osaka, Japan; 5 Section of Biological Safety Research, Tama Laboratory, Japan Food Research Laboratories, Tama, Tokyo, Japan; University of Texas Southwestern Medical Center, UNITED STATES

## Abstract

Osteoporosis with bone loss is widely recognized as a major health problem. Bone homeostasis is maintained by balancing bone formation and bone resorption. The imbalance caused by increased bone resorption over bone formation can lead to various bone-related diseases such as osteoporosis and rheumatoid arthritis. Osteoclasts are the principal cells responsible for bone resorption and the main targets of anti-resorptive therapies. However, excessive inhibition of osteoclast differentiation may lead to inhibition of osteoblast differentiation. Therefore, it is important to screen for new compounds capable of inhibiting bone resorption and enhancing bone formation. *Toddalia asiatica* (L.) Lam. has been utilized traditionally for medicinal purposes such as the treatment of rheumatism. Currently, the extract is considered to be a good source of pharmacological agents for the treatment of bone-related diseases, but the active compounds have yet to be identified. We investigated whether toddaculin, derived from *Toddalia asiatica* (L.) Lam., affects both processes by inhibiting bone resorption and enhancing bone formation. Towards this end, we used pre-osteoclastic RAW 264 cells and pre-osteoblastic MC3T3-E1 cells. We found that toddaculin not only inhibited the differentiation of osteoclasts via activation of the NF-κB, ERK 1/2, and p38 MAPK signaling pathways, but it also induced differentiation and mineralization of osteoblasts by regulating differentiation factors. Thus, toddaculin might be beneficial for the prevention and treatment of osteoporosis.

## Introduction

Osteoclasts resorb bone and support normal bone remodeling. Osteoclasts are regulated by receptor activator of nuclear factor kappa B (NF-κB) ligand (RANKL) and macrophage colony-stimulating factor (M-CSF) [1]. Since tartrate-resistant acid phosphatase (TRAP) is highly expressed in osteoclasts, TRAP activity is used as a sensitive marker for osteoclasts [2]. Excess osteoclast activity is known to lead to various bone-related diseases such as osteoporosis and rheumatoid arthritis [3]. On the other hand, osteoblasts stimulate mineralization and bone production by regulating proliferation and differentiation of osteoblast precursors [4]. Alkaline phosphatase (ALP) is highly expressed in osteoblasts, and thus ALP activity is used as a sensitive marker for osteoblasts [5].

Previous therapies for bone-related diseases have focused on inhibition of bone resorption. Besides inhibition of bone resorption, recent therapies have targeted bone formation [6]. Therefore, screening of natural compounds capable of both effects (inhibition of bone resorption and enhancement of bone formation) might be a useful approach in the treatment of bone-related diseases.

**Fig 1:**
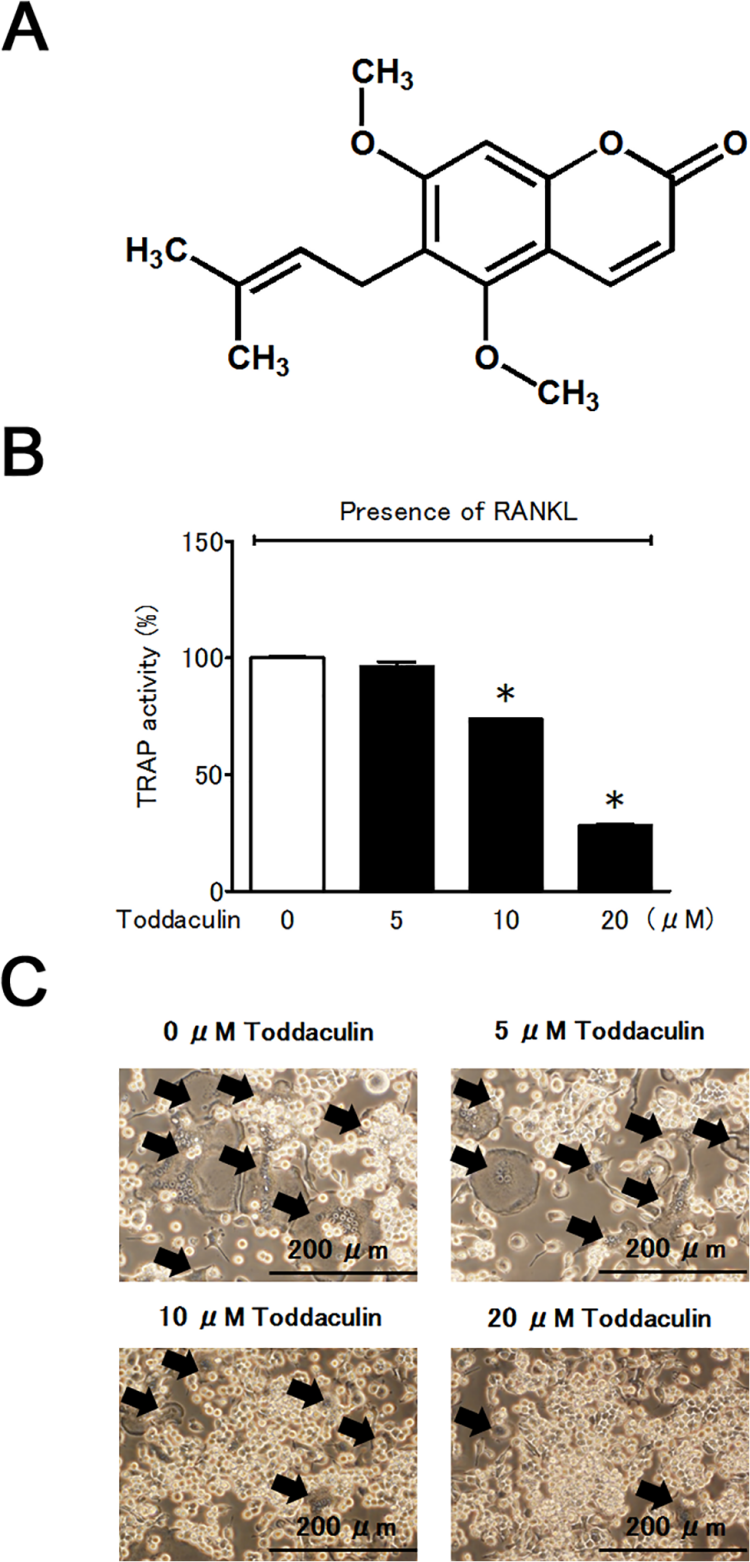
Effect of toddaculin on osteoclastogenesis. Chemical structure of toddaculin (A) and its effect on TRAP activity in RANKL-treated RAW 264 cells (B). Values are expressed as means ± SEM (n = 3). *, *P* < 0.05 compared with control. Light microscopic analysis of osteoclast formation is shown in (C). Arrows show multi-nucleated cells. Each picture is representative of at least triplicate analyses.

*Toddalia asiatica* (L.) Lam. (*T*. *asiatica*) belongs to the family *Rutaceae*, and is widely recognized as a medicinal plant in Africa, India, China and Japan. *T*. *asiatica* has been used for the treatment of many diseases including rheumatism [7–9]. However, the active agents of *T*. *asiatica* and their mechanisms of action are still unclear. Recently, Ramiro *et al*. isolated toddaculin (Fig 1A) from the stem bark of *T*. *asiatica* and showed that the compound decreased phosphorylation of extracellular signal-regulated kinases 1 and 2 (ERK 1/2) and mitogen-activated protein kinase (MAPK) in U-937 cells [10]. Since the ERK 1/2 pathway is reportedly involved in negative regulation of skeletal mineralization, toddaculin may not only suppress excess osteoclast activity but also enhance osteoblast differentiation and mineralization [11].

Based on this background, in the present study, we investigated whether toddaculin had both effects (inhibition of bone resorption and enhancement of bone formation) by *in vitro* studies of pre-osteoclastic RAW 264 cells and pre-osteoblastic MC3T3-E1 cells.

## Materials and Methods

### Reagents and cells

Alpha-minimum essential medium eagle (α-MEM) and penicillin-streptomycin were purchased from Sigma-Aldrich Japan (Tokyo, Japan). Fetal bovine serum (FBS) and GlutaMAX were purchased from Life Technologies Japan (Tokyo, Japan). Recombinant soluble mouse RANK ligand (RANKL) was from R&D Systems (Minneapolis, USA). Ascorbic acid and β-glycerophosphate were purchased from Wako Pure Chemical (Osaka, Japan). All other reagents used were of analytical grade.

RAW 264 and MC3T3-E1 murine cell lines were obtained from the RIKEN Cell Bank (Tsukuba, Japan). These cells were cultured in α-MEM supplemented with 10% FBS, 1% GlutaMAX, penicillin (100 units/mL) and streptomycin (100 μg/mL) in an atmosphere of 5% CO_2_ at 37°C.

### Isolation of toddaculin and preparation of experimental medium

Toddaculin was isolated from 100 g of dry stem chips (Okinawa Medical Herb Association (Okinawa, Japan)) as described previously [8]. The purity of the toddaculin used in this study was > 95% as determined by HPLC. Toddaculin was dissolved in dimethyl sulfoxide (DMSO). The DMSO solution was diluted with experimental medium (10% FBS/1% GlutaMAX/α-MEM containing 10 ng/mL RANKL, 50 μg/mL ascorbic acid or 10 mM β-glycerophosphate) to achieve the desired final concentration of 0–40 μM toddaculin. The final concentration of DMSO in the experimental medium was less than 0.1% (v/v) and did not affect cell viability. Medium containing DMSO alone was prepared similarly and used as the control medium.

### Osteoclast differentiation and TRAP activity of RAW 264 cells

RAW 264 cells (2.5 × 10^4^ cells/well) were cultured in 24-well plates and incubated for 24 h. Cells were then incubated in the experimental medium containing 0–20 μM toddaculin and 10 ng/mL RANKL for an additional 48 h. Then, the cells were collected, washed with saline and treated with lysis buffer (0.1 M Tris-HCl pH 7.5 containing 0.1% Triton-X 100). TRAP activity in the cell lysate was measured using a TRACP and ALP assay kit (Takara Bio, Shiga, Japan) according to the manufacturer’s protocol. Light microscopic analysis was performed with an Olympus IX71 microscope (Tokyo, Japan) at 200 × magnification.

### ALP activity of MC3T3-E1 cells

MC3T3-E1 cells (5.0 × 10^4^ cells/well) were cultured in 24-well plates and incubated for 7 days. Cells were then incubated in the experimental medium containing 0–40 μM toddaculin and 50 μg/mL ascorbic acid for an additional 4 days. During the incubation, the experimental medium was changed every 2 days. After that, the cells were collected, washed with saline and treated with lysis buffer (0.1 M Tris-HCl pH 7.5 containing 0.1% Triton-X 100). ALP activity in the cell lysate was evaluated using a TRACP and ALP assay kit (Takara Bio).

### DNA microarray and mRNA analysis

RAW 264 cells (2.5 × 10^4^ cells/well) were incubated in the experimental medium containing 0–20 μM toddaculin and 10 ng/mL RANKL for 48 h. MC3T3-E1 cells (5.0 × 10^4^ cells/well) were cultured in the experimental medium containing 0–40 μM toddaculin and 50 μg/mL ascorbic acid for 4 days. After incubation, total RNA was extracted from the cells using an RNeasy Mini Kit (Qiagen, Tokyo, Japan). DNA microarray analysis was performed using the Genopal Mouse Bone Metabolism Chip (Mitsubishi Rayon, Tokyo, Japan) according to the manufacturer’s protocol.

For real-time quantitative reverse-transcription PCR (real-time RT-PCR), cDNAs were synthesized using random primers and PrimeScript Reverse Transcriptase (Takara Bio). The target cDNAs were amplified using Fast SYBR Green Master Mix (Life Technologies, Japan) together with gene-specific primers (Takara Bio) (Table 1). PCR conditions were 95°C for 20 s, 95°C for 3 s, and 60°C for 30 s for 40 cycles. PCR products were measured using a StepOnePlus Real-time PCR System (Life Technologies Japan). The fold-change expression analysis was performed using a comparative Cq method (2-delta-delta Cq) [12]. *Actb* was used as a reference gene.

**Table 1:** Primer pairs used for RT-PCR.

GenBank ID	Gene name	Primer	Primer sequence (5′ to 3′)
NM_007393	*Actb*	Forward	CATCCGTAAAGACCTCTATGCCAAC
		Reverse	ATGGAGCCACCGATCCACA
NM_001102405	*Trap*	Forward	GTGCTGGCTGGAAACCATGA
		Reverse	GTCCAGCATAAAGATGGCCACA
NM_007802	*Ctsk*	Forward	CACCCAGTGGGAGCTATGGAA
		Reverse	GCCTCCAGGTTATGGGCAGA
NM_175632	*Oscar*	Forward	CGTGCTGACTTCACACCAACA
		Reverse	AAGGTCACGTTGATCCCAGGAG
NM_013599	*Mmp9*	Forward	GCCCTGGAACTCACACGACA
		Reverse	TTGGAAACTCACACGCCAGAAG
NM_008317	*Hyal1*	Forward	CCGTAATGCCCTACGTCCAG
		Reverse	AAGGGCCCAAGTGTGGAA
NM_010235	*Fosl1*	Forward	ACCGGTCCACAGAGGTTCAT
		Reverse	GCCTCTCGGAGTCTGGTCTT
NM_007431	*Alpl*	Forward	GCAGTATGAATTGAATCGGAACAAC
		Reverse	ATGGCCTGGTCCATCTCCAC
NM_007541	*Bglap*	Forward	TCTCTGACCTCACAGATGCCAAG
		Reverse	AGCGCCGGAGTCTGTTCACTA

### NF-κB luciferase reporter gene assay of RAW 264 cells

RAW 264 cells were transfected with firefly pGL4.32/NF-κB reporter plasmid and *Renilla* pGL4.73/SV40 control plasmid (Promega, Tokyo, Japan) using Lipofectamine LTX and PLUS Reagents (Life Technologies Japan) according to the manufacturer’s protocol. Transfected RAW 264 cells (5.0 × 10^4^ cells/well) were cultured in 96-well plates and incubated for 16 h. Cells were then incubated in the experimental medium containing 0–20 μM toddaculin and 10 ng/mL RANKL for an additional 5 h. Firefly luciferase and *Renilla* luciferase activities were evaluated with the Dual-Glo Luciferase Assay System (Promega). The values were normalized by Renilla luciferase activities.

### Western blot analysis of RAW 264 cells

RAW 264 cells (5.0 × 10^6^ cells/flask) were cultured in 25 cm^2^ flasks. The cells were then incubated in the experimental medium containing 20 μM toddaculin and 10 ng/mL RANKL for an additional 0, 2 and 4 h. Then, the cells were lysed using Complete Lysis M and Phos-STOP (Roche Diagnostics, Tokyo, Japan). Lysates were centrifuged at 13,000 rpm for 5 min, and supernatants were collected. Protein concentrations in the supernatants were measured. Equal amounts of protein were separated by SDS-PAGE and transferred to a PVDF membrane (Merck Millipore, MA, USA). The membrane was blocked with ECL Blocking reagent (GE Healthcare, Tokyo, Japan) and incubated with primary antibodies (anti-β-actin, anti-p38, anti-phospho-p38, anti-ERK 1/2, anti-phospho-ERK 1/2, anti-c-Jun N-terminal protein kinase (JNK) or anti-phospho-JNK rabbit polyclonal antibodies (Cell Signaling Japan, Tokyo, Japan)) and HRP-linked secondary antibodies (GE Healthcare). The membranes were developed using ECL Prime Western Blotting Detection Reagents (GE Healthcare) and imaged on an LAS-4000 Luminescent Image Analyzer (GE Healthcare). Band intensities were measured for statistical analysis using Multi Gauge, version 3.11, software (Fujifilm Life Science, Tokyo, Japan).

### Mineralization assay of MC3T3-E1 cells

MC3T3-E1 cells (2.5 × 10^4^ cells/well) were cultured in 24-well plates and incubated for 7 days. Cells were then incubated in the experimental medium containing 0–40 μM toddaculin, 50 μg/mL ascorbic acid and 10 mM β-glycerophosphate for an additional 14 days. During the incubation, the experimental medium was changed every 2 days. After that, the cells were fixed with ice-cold 70% ethanol and stained with Alizarin red. For quantification, cells stained with Alizarin red were destained with cetylpyridinium chloride, then the extracted stain was transferred to 96-well plates, and the absorbance at 550 nm was measured using a microplate reader [13]. Light microscopic analysis was performed at 400 × magnification with an Olympus IX71 microscope.

### Statistical analysis

Data are expressed as means ± standard error of the mean (SEM). Unless otherwise noted, each value was the result of three independent experiments (n = 3). Comparisons between groups were analyzed using one-way analysis of variance followed by Dunnett’s multiple comparisons test or two-tailed unpaired Student's t test using GraphPad Prism V5.02 software for Windows (GraphPad Software, San Diego, California, USA). *P*-values < 0.05 were considered significant.

## Results

### Toddaculin suppressed RANKL-induced osteoclast activity in RAW 264 cells

The effect of toddaculin on osteoclast differentiation of RAW 264 cells was investigated. Toddaculin, isolated from *T*. *asiatica*, did not affect cell proliferation at concentrations up to 20 μM in the presence of RANKL (S1 Fig), but it inhibited TRAP activity and suppressed formation of multi-nucleated cells in a dose-dependent fashion (Fig 1B and 1C). These results indicated that toddaculin inhibited differentiation of pre-osteoclastic RAW 264 cells into osteoclast-like cells, especially in the presence of RANKL.

### Effect of toddaculin on mRNA expression in RAW 264 cells

**Fig 2:**
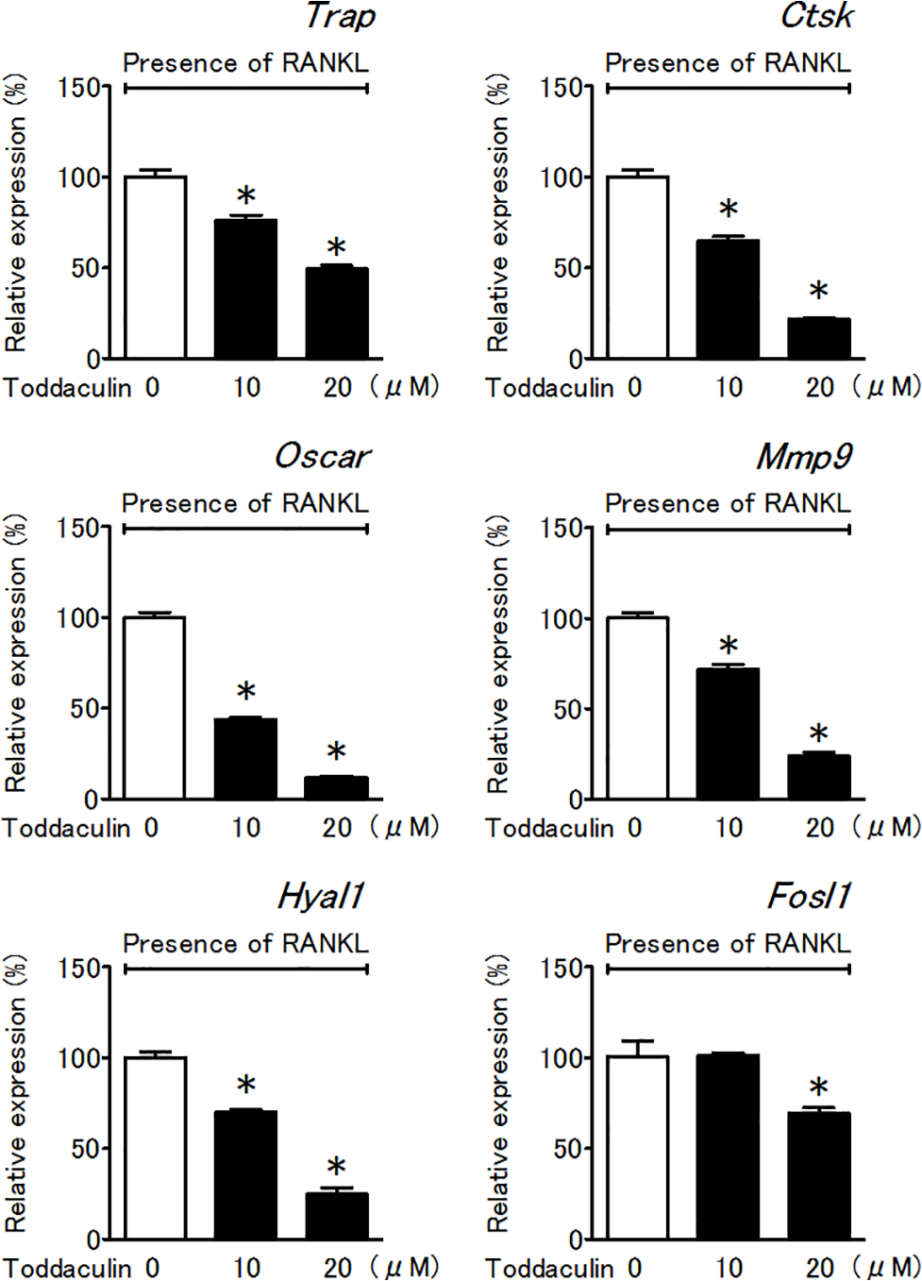
Effect of toddaculin on mRNA expression of osteoclast-related genes (*Trap*, *Ctsk*, *Oscar*, *Mmp9*, *Hyal1*, and *Fosl1*) in RANKL-treated RAW 264 cells. Values are expressed as means ± SEM (n = 3). *, *P* < 0.05 compared with control.

To investigate the mechanism by which toddaculin inhibited osteoclast differentiation of RAW 264 cells, we evaluated their gene expression profile. DNA microarray analysis showed that 20 μM toddaculin treatment led to downexpression of osteoclast-related genes (*Trap*, Cathepsin K (*Ctsk*), osteoclast associated receptor (*Oscar*), matrix metalloproteinase 9 (*Mmp9*), hyaluronoglucosaminidase 1 (*Hyal1*) and FOS-like antigen 1 (*Fosl1*)) (data not shown). We then evaluated these genes with real-time RT-PCR and confirmed that expression of these genes was decreased by toddaculin in a dose-dependent fashion (Fig 2). Therefore, it appeared that toddaculin inhibited RAW 264 cell differentiation via effects on mRNA expression related to the osteoclastogenic response. On the other hand, in this study, *Fosl1* did not seem to be as affected by toddaculin as other genes, probably due to rather low induction of *Fosl1* by RANKL.

### Toddaculin inhibited RANKL-induced NF-κB activation and MAPKs phosphorylation in RAW 264 cells

**Fig 3:**
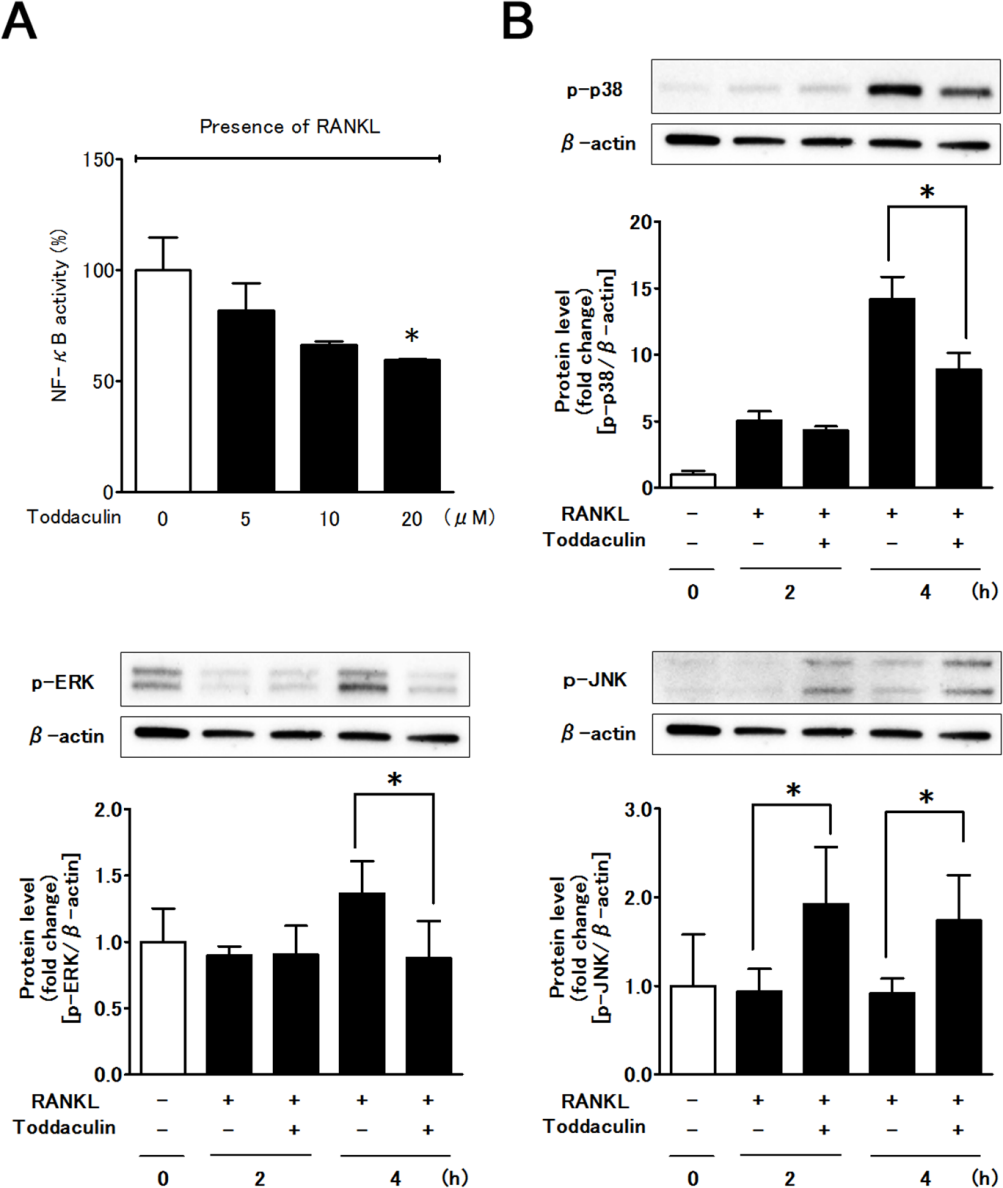
Effect of toddaculin on RANKL-induced NF-κB activation (A) and MAPKs phosphorylation (B) in RAW 264 cells. Values are expressed as means ± SEM (n = 3 for NF-κB activation and n = 4 for MAPKs phosphorylation). *, *P* < 0.05 compared with control.

To explore pathways by which toddaculin regulated osteoclast differentiation and the function of RAW 264 cells, we performed luciferase assays and found that toddaculin inhibited NF-κB activity in a concentration-dependent manner in the presence of RANKL (Fig 3A). In Western blot analysis, toddaculin inhibited RANKL-induced phosphorylation of p38 and ERK 1/2 MAPK (Fig 3B). The levels of p38, JNK, and ERK 1/2 MAPK were not affected by toddaculin (S2 Fig). The phosphorylation level of JNK was unaffected by RANKL treatment, and its level was increased by 20 μM toddaculin. Thus, the anti-osteoclastogenic effect of toddaculin could be attributed to disruption of NF-κB, p38, and ERK 1/2 signaling. Contribution of JNK activation to the anti-osteoclastogenic effect of toddaculin is unclear, and further study is needed.

### Toddaculin increased ALP activity in MC3T3-E1 cells

**Fig 4:**
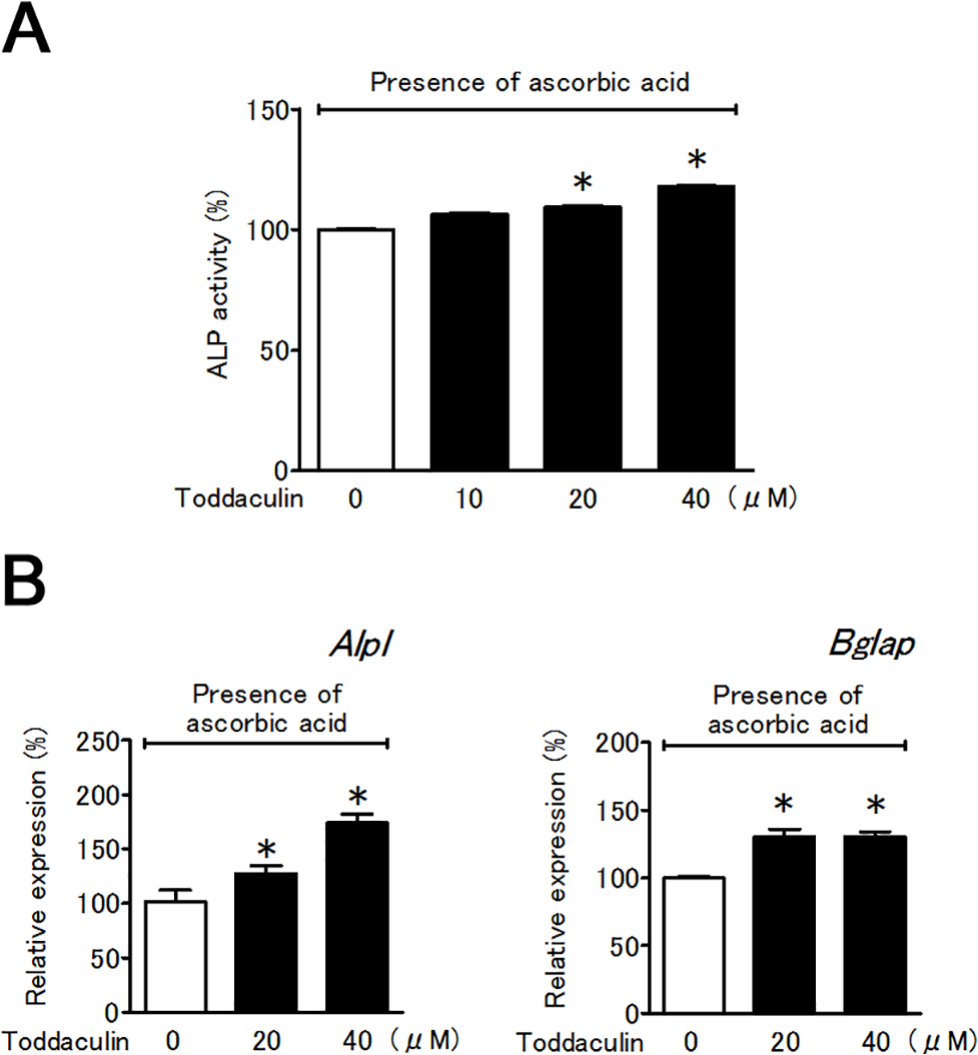
Effect of toddaculin on ALP activity (A) and osteoblast-related genes (*Alpl* and *Bglap*) (B) in ascorbic acid-treated MC3T3-E1 cells. Values are expressed as means ± SEM (n = 3). *, *P* < 0.05 compared with control.

Next, we investigated the effect of toddaculin on osteoblast differentiation of MC3T3-E1 cells. Toddaculin enhanced cellular ALP activity in a dose-dependent manner in the presence of ascorbic acid (Fig 4A) without affecting cell proliferation (S3 Fig). These data suggested that toddaculin was a unique compound that not only inhibited differentiation of RAW 264 cells but also enhanced osteoblast differentiation by MC3T3-E1 cells.

### Effect of toddaculin on mRNA expression in MC3T3-E1 cells

DNA microarray analysis showed that 20 μM toddaculin increased the expression of osteoblast-related genes (*Alpl* and osteocalcin (*Bglap*)) (data not shown). The results were further supported by data from real-time RT-PCR (Fig 4B). Hence, it is likely that toddaculin enhanced MC3T3-E1 differentiation via regulation of osteoblast-related genes.

### Toddaculin enhanced bone nodule formation by MC3T3-E1 cells

**Fig 5:**
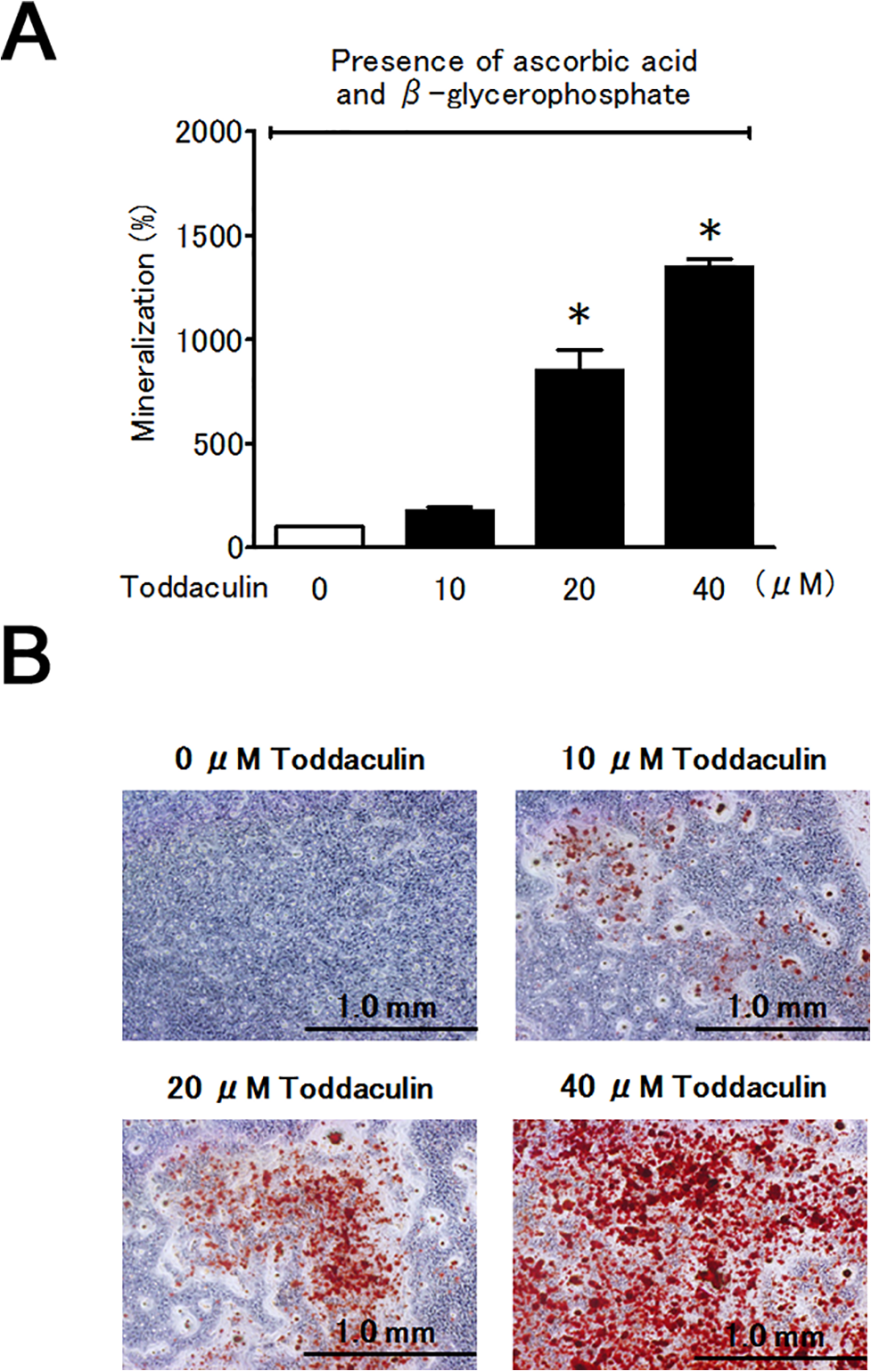
Effect of toddaculin on osteoblast mineralization in ascorbic acid and β-glycerophosphate-treated MC3T3-E1 cells that were evaluated by Alizarin red S staining (microplate reader analysis (A) and light microscopic evaluation (B)). Values are expressed as means ± SEM (n = 3). *, *P* < 0.05 compared with control. Each picture is representative of at least triplicate analyses.

Finally, we investigated the effect of toddaculin on bone matrix mineralization by MC3T3-E1 cells. Toddaculin significantly enhanced bone nodule formation in a concentration-dependent manner as evaluated by Alizarin red S staining (Fig 5A and 5B). These results indicated that toddaculin enhanced mineralized nodule formation by osteoblasts.

## Discussion

Osteoporosis with bone loss is widely recognized as a major health problem afflicting more than 200 million people worldwide [14, 15]. Current treatment options for post-menopausal osteoporosis target either the osteoclast (i.e., anti-resorptive bisphosphonates and Denosumab) or the osteoblast (i.e., anabolic parathyroid hormone) [16]. However, the uncoupling of bone remodeling by targeting only one of these cell populations has led to much concern in the field [17]. Therefore, inquiry into natural options that have the potential to impact both the osteoblast and the osteoclast are deemed a promising therapeutic alternative.

Toddaculin, which belongs to the chemical class of coumarins, is one of the major bioactive components of *T*. *asiatica*. Nakamura *et al*. showed that toddaculin suppressed lipopolysaccharide (LPS)-induced nitric oxide (NO) production [18]. LPS is a bone resorption-inducing factor and known to be activated through adaptor protein tumor necrosis factor receptor-associated factor 6 (TRAF6) signaling. LPS-induced TRAF6 signaling is similar to that induced by RANKL, and TRAF6 signaling leads to activated NF-κB and MAPKs (ERK 1/2, p38, and JNK) [19, 20]. Ramiro *et al*. reported that toddaculin decreased phosphorylation of ERK 1/2 MAPK in U-937 cells [10]. Kono *et al*. showed that the ERK 1/2 pathway was involved in negative regulation of skeletal mineralization [11]. Considering these studies [10, 11, 18–20], toddaculin may not only suppress excess osteoclast activity but also enhance osteoblast differentiation and mineralization. However, no data are available regarding the effects of toddaculin on bone-related diseases.

In this study, we asked whether toddaculin was capable of inhibiting bone resorption and enhancing bone formation. Thus, we analyzed pre-osteoclastic RAW 264 cells and pre-osteoblastic MC3T3-E1 cells. First, we examined the effect of toddaculin on RANKL-induced osteoclastogenesis using RAW 264 cells and found that toddaculin effectively inhibited TRAP activity and suppressed formation of multi-nucleated cells. Osteoclastogenesis was accompanied by expression of various transcription factors and osteoclast-specific genes [21, 22]. We therefore analyzed expression of these genes using DNA microarray analysis and real-time RT-PCR. We found that the expression of several genes (*Trap*, *Ctsk*, *Oscar*, *Mmp9*, *Hyal1* and *Fosl1*) was attenuated by toddaculin. These results clearly suggested that toddaculin was capable of attenuating RAW 264 differentiation into osteoclast-like cells.

**Fig 6:**
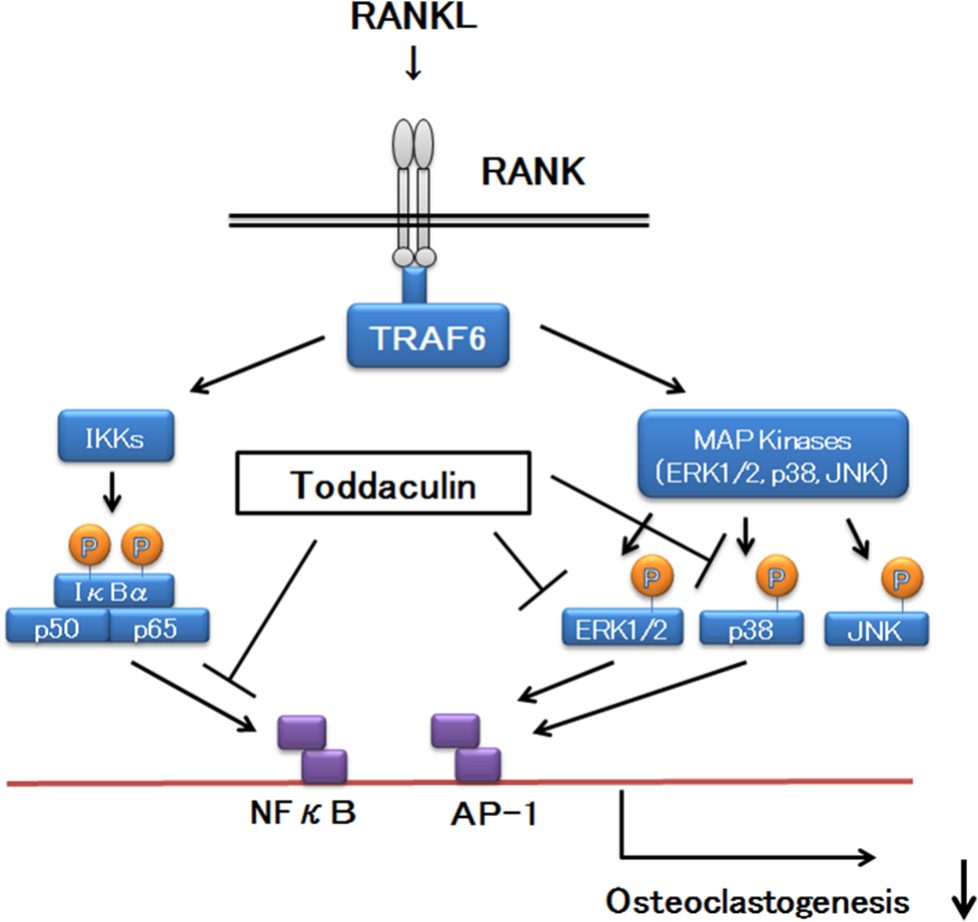
A proposed mechanism of the inhibitory effects of toddaculin on osteoclastogenesis.

When RANKL binds to RANK, many signaling pathways (e.g., NF-κB and MAPKs) are rapidly activated through adaptor protein TRAF6 signaling [1, 19]. Signaling by NF-κB and MAPKs (ERK 1/2, p38, and JNK) plays an important role in osteoclastogenesis. Suppression of the signaling is thought to inhibit osteoclast differentiation. Interestingly, in the present study, toddaculin was shown to reduce RANKL-induced NF-κB activity and inhibit phosphorylation of p38 and ERK 1/2, but not JNK. Therefore, the anti-osteoclastogenic effects of toddaculin were mainly caused by disrupting NF-κB, p38 and ERK 1/2 signaling. A proposed mechanism for the inhibitory effects of toddaculin on osteoclastogenesis is shown in Fig 6. The effects of toddaculin on JNK phosphorylation and its contribution to anti-osteoclastogenic activity are unclear. Since it is known that JNK phosphorylation is activated by ER stress, further study is needed to evaluate the effects and mechanism, including ER stress by toddaculin [23].

The expression of osteocalcin, osteopontin and ALP in osteoblasts promotes osteoblast proliferation, which leads to new bone formation [24–26]. We examined the effect of toddaculin on osteoblastogenesis using MC3T3-E1 cells and found that toddaculin promoted ALP activity and increased osteoblastic marker genes (*Alpl* and *Bglap*). ALP is known to be critically involved in the initiation of mineralization during bone formation. Osteocalcin appears to act during the terminal phase of osteoblastic differentiation [24]. Thus, these effects of toddaculin on ALP activity and osteoblast-related genes (*Alpl* and *Bglap*) indicate the ability of toddaculin to promote osteoblastogenesis. Since toddaculin enhanced the formation of calcium deposits, toddaculin is capable of enhancing MC3T3-E1 differentiation into osteoblast-like cells. But, it is possible that the increase in osteoblastogenesis might result in an increase in RANKL, which would in turn increase osteoclastogenesis *in vivo*. A recent study of osteoblastogenesis reported that NF-κB inhibition led to bone formation, thereby exhibiting a protective effect against bone loss [27]. Meanwhile, the role of the MAPK signaling pathway in osteoblastogenesis is still unclear. The importance of JNK signaling for late-stage osteoblast differentiation has recently been reported [28]. Hence, the effect of toddaculin on NF-κB, MAPK, and JNK is intriguing. Further study is needed to evaluate the complex relationships outlined above.

As a point of reference, in the present study, the osteoblast experiments required a higher concentration of toddaculin than the osteoclast experiments. This may be due to the different numbers of cells and different cell strains used in the experiments. In conclusion, we identified toddaculin from *T*. *asiatica* as a potential compound for bone disease treatment. Toddaculin not only inhibited differentiation of osteoclasts via activation of NF-κB, ERK 1/2, and p38 MAPK signaling pathway, but also induced differentiation and mineralization of osteoblasts by regulating differentiation factors. To the best of our knowledge, this is the first study to determine the potential of toddaculin for bone regeneration applications. This therapy might be beneficial for the prevention and treatment of osteoporosis. Further studies, especially those performed *in vivo*, are needed to better understand the function of toddaculin within the body.

## Supporting Information

S1 Fig Effects of toddaculin on cell proliferation of RANKL-treated RAW 264 cells. RAW 264 cells were treated with toddaculin in the presence of RANKL for 48 h. After that, cell proliferation was assessed by WST-8 reagent [29].Values are expressed as means ± SEM (n = 3).
(TIF)

S2 Fig Effect of toddaculin on RANKL-induced MAPKs phosphorylation in RAW 264 cells. Western blot represents at least three separate experiments.
(TIF)

S3 Fig Effects of toddaculin on cell proliferation in ascorbic acid-treated MC3T3-E1 cells. MC3T3-E1 cells were treated with toddaculin in the presence of ascorbic acid for 4 days. After that, cell proliferation was assessed by WST-8 reagent [29]. Values are expressed as means ± SEM (n = 3).
(TIF)
