# Supplementary figures and images for: Toddaculin, Isolated from of Toddalia asiatica (L.) Lam., Inhibited Osteoclastogenesis in RAW 264 Cells and Enhanced Osteoblastogenesis in MC3T3-E1 Cells
Source: PLoS One. 2015 May 18;10(5):e0127158. doi: 10.1371/journal.pone.0127158 (PMC4436367; doi:10.1371/journal.pone.0127158)

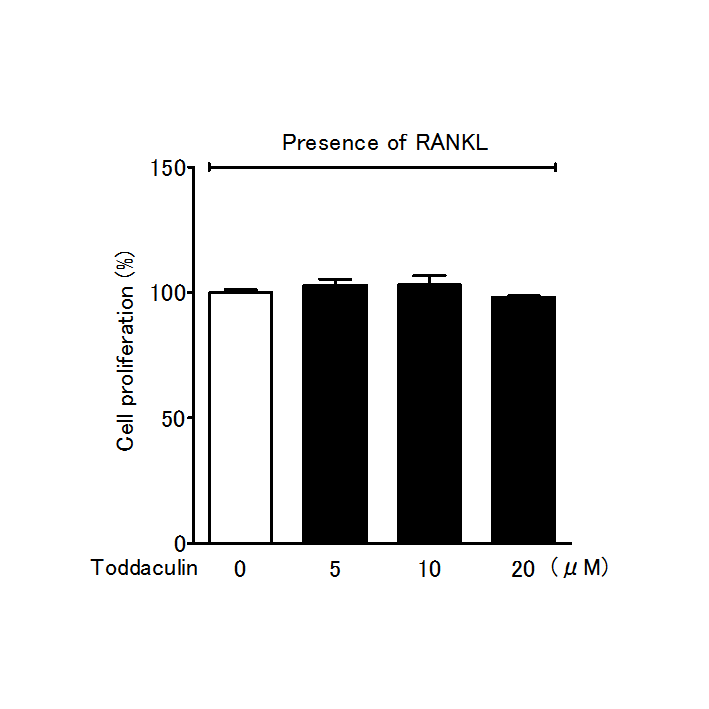

Supplement: S1 Fig — RAW 264 cells were treated with toddaculin in the presence of RANKL for 48 h. After that, cell proliferation was assessed by WST-8 reagent [29].Values are expressed as means ± SEM (n = 3). (TIF) [file pone.0127158.s001.tif]

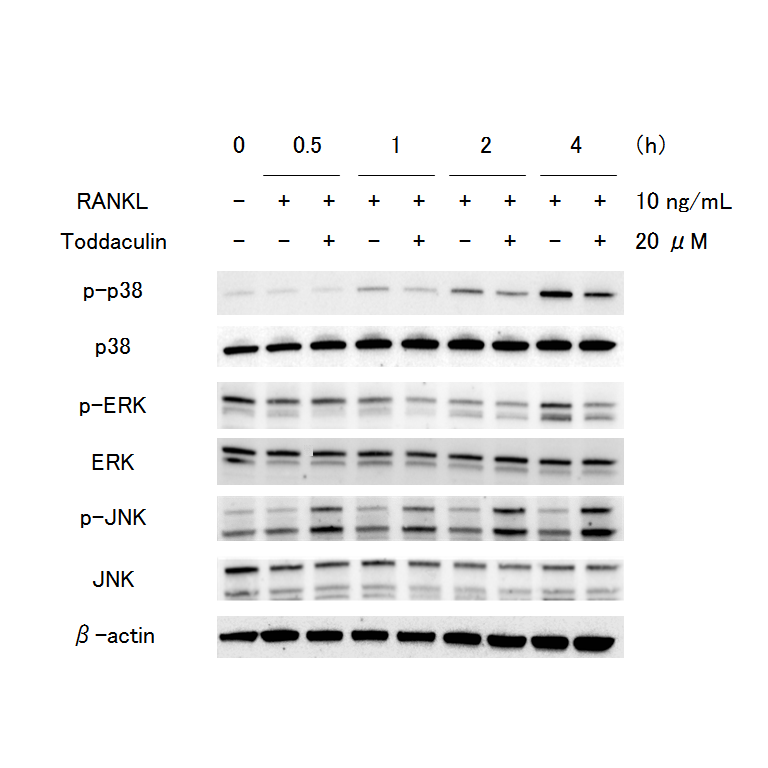

Supplement: S2 Fig — Western blot represents at least three separate experiments. (TIF) [file pone.0127158.s002.tif]

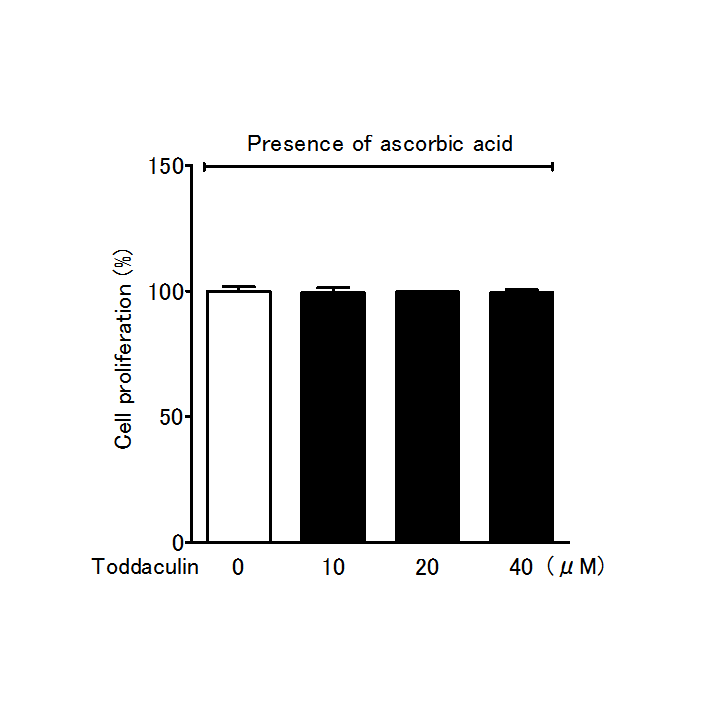

Supplement: S3 Fig — MC3T3-E1 cells were treated with toddaculin in the presence of ascorbic acid for 4 days. After that, cell proliferation was assessed by WST-8 reagent [29]. Values are expressed as means ± SEM (n = 3). (TIF) [file pone.0127158.s003.tif]
